# A Material–Process–Equipment Integrated Design Method for Accelerating the Process Development of Twin-Screw Wet Granulation

**DOI:** 10.3390/ph19060921

**Published:** 2026-06-11

**Authors:** Liping Chen, Wuzhen Qi, Juntao Xie, Yidan Wang, Shuying Zhao, Xiao Ma, Yifan Hu, Hui Jiang, Ying Liu, Bing Xu

**Affiliations:** 1 Department of Chinese Medicine Informatics, Beijing University of Chinese Medicine, Beijing 100029, China; 20230935129@bucm.edu.cn (L.C.); wuzhen.qi@bucm.edu.cn (W.Q.); 20240935221@bucm.edu.cn (J.X.); 20240935146@bucm.edu.cn (Y.W.); 20230935202@bucm.edu.cn (S.Z.); 20230935203@bucm.edu.cn (X.M.); 20240935222@bucm.edu.cn (Y.H.); 20240935282@bucm.edu.cn (H.J.); 2 Beijing Research Institute of Chinese Medicine, Beijing University of Chinese Medicine, Beijing 102488, China; 3 Beijing Key Laboratory of Chinese Medicine Manufacturing Process Control and Quality Evaluation, School of Chinese Materia Medica, Beijing University of Chinese Medicine, Beijing 100029, China

**Keywords:** twin-screw wet granulation, material-process-equipment integrated design, equivalent formulation, critical screw parameters, 3D printing

## Abstract

**Background**: Twin-screw wet granulation (TSWG) is a promising continuous manufacturing technology, featuring high operational flexibility, short residence time and consistent quality. The process development of TSWG relies on the synergy of material characterization, screw configuration, and process parameter optimization. **Objective**: In order to fully combine various design variables, and to accelerate the process development of TSWG, a material–process–equipment integrated design (MPEID) methodology is first applied to the TSWG process of Guizhi Fuling capsule, a botanical drug product. **Methods**: First, an equivalent formulation was designed to save trial costs. Second, 3D printing technology was used to customize both conveying and kneading elements with the lead, with the kneading discs stagger angle (*SA*) and the thickness (*thick*) as screw element variables. The position of fabricated kneading elements was varied to generate different screw configurations. Then, the critical screw parameters (CSPs) and critical process parameters (CPPs) were identified by a two-step design of experiment (DOE) toward optimizing granule quality. **Results**: As a result, the *SA* and *thick* were identified as CSPs, and the liquid-to-solid ratio was the CPP. Under the optimal TSWG process conditions, the twin-screw granulator could be operated under low torque (i.e., average torque = 1.48 ± 0.06 Nm). The dried granules exhibited superior flowability, as well as highly consistent particle size distribution with industrial batches. After capsule filling, the dissolution test results showed the prepared Guizhi Fuling capsules reached 93.7% cumulative dissolution at 15 min, which approached that of commercial capsules (i.e., 93.0%). **Conclusions**: This study demonstrated the feasibility of proposed MPEID methodology, supporting the efficient and cost-effective process development of TSWG.

## 1. Introduction

Over the past decade, continuous manufacturing of oral solid dosage forms has become a central trend in pharmaceutical modernization [1,2]. To date, 17 drug products developed through continuous manufacturing have been approved by the FDA [3,4,5]. Twin-screw wet granulation (TSWG), as an advanced continuous granulation technology, has attracted considerable attention for the development of oral solid dosage forms. TSWG offers the advantages of high throughput, short material residence time, flexible equipment design, and superior process controllability [6,7]. Under the Quality by Design (QbD) framework, the process development of TSWG has focused not only on the effects of critical material properties (CMAs, e.g., formulation composition or binder type) [8] and critical process parameters (CPPs, e.g., liquid-to-solid (*L/S*) ratio or screw speed) [9] on critical quality attributes (CQAs) [10,11,12,13], but also on the effects of critical screw parameters (CSPs) on CQAs.

So far, most studies on TSWG process development have evaluated the interactions among CQAs, CMAs, and CPPs under a predefined, fixed screw configuration. Research has shown that the *L/S* ratio remains one of the most critical process parameters governing granule properties. Increasing the *L/S* ratio strengthened liquid bridges, enlarged particle size, and shifted the particle size distribution (PSD) from bimodal to unimodal, as validated across formulations containing different APIs [14,15,16]. The screw speed and feed rate were not studied independently; rather, they were linked to barrel filling degree and the residence time distribution to quantitatively explain their effects on torque fluctuation, granule porosity, and mechanical strength [17,18,19]. At the same throughput, a higher screw speed reduced the barrel filling and torque while shortening the residence time. In contrast, a higher feed rate increased the battel filling degree and shear intensity, producing denser granules [18,19]. Powder physical properties (i.e., wettability, bulk density and swelling behavior) interacted with process parameters to determine granulation performance. The same process parameters often led to different results across formulations [20,21,22].

Unlike process parameters controlled by automation systems, the screw element configuration, including element type, geometric parameters and assembly sequence, is a modifiable factor in TSWG and can be varied from a pure machine setup viewpoint. The conveying elements (CEs) and kneading elements (KEs) are two core functional components in TSWG screws, and different combinations of screw elements produce granules with diverse characteristics [23], such as granule shape [17,24], size [25,26,27,28,29], porosity [30,31] and flowability [32,33], etc. For CEs, the key geometric parameters, including thread lead or pitch, directly govern the PSD of granules, as well as the ratio of fines to oversized granules [14,31,34]. Conveying elements facilitate rapid material transport and reduce overall residence time. A shorter pitch or lead increases local material retention and shear frequency, whereas a longer pitch accelerates transport and shortens wetting duration [31,34]. Insufficient wetting time restricts liquid spreading and leads to fragile liquid bridges between particles [34]. Peeters et al. [14] demonstrated that conveying element geometry interacted with material wettability to determine granule porosity. A wider pitch yields higher porosity because of limited liquid penetration and weak interparticle bonding. A narrower pitch improves liquid spreading and reduces granule porosity via more effective compaction. Previous studies have shown that using only CEs could lead to insufficient mixing and compaction. This results in high-porosity granules, excessive fines, and an undesirable bimodal PSD [35], thereby compromising product uniformity and stability [13,23].

In contrast to CEs, KEs improve granule density and uniformity through targeted compaction and fragmentation. They are among the most widely studied functional elements in TSWG screw design [25,32]. Increasing the stagger angle from 30° to 60° prolongs local residence time and strengthens shear intensity. This promotes uniform liquid spreading and stable liquid bridge formation [36]. Wider kneading discs favor dispersive mixing and granule densification, whereas narrower discs enhance distributive mixing and liquid distribution [37]. Increasing the number of kneading discs from 4 to 12 further extends residence time and substantially improves liquid–powder mixing uniformity. This effectively reduces fine particle generation and optimizes granule size distribution [38]. Kiricenko [39] systematically investigated the effects of KE geometric parameters on the quality of granules and tablets, including 1–2 kneading zones, 3–11 kneading discs, disc thickness of 1.4–5.4 mm, and stagger angles of 30–90°. Increasing disc thickness, disc count or stagger angle increases process torque and prolongs residence time. This significantly improves liquid distribution uniformity and yields denser granules with lower friability. It is confirmed that at least one kneading zone is essential in the screw configuration. A rational KE design improves liquid distribution substantially and produces granules and tablets with low friability and adequate mechanical strength.

Notwithstanding these advances, major limitations remain in the screw configuration research on TSWG. First, most studies still rely on empirical approaches to evaluate and optimize screw configurations. Most studies investigated CE and KE factors separately, and few have explored their synergistic effects [26,27,28]. Second, the vendor-supplied modules of exchangeable screw elements were often limited, which may restrict the screw design space that can be adapted for development purposes to fit the formulation and process. The 3D printing technology provides a cost-effective solution for the rapid design, fabrication and functional validation of complex screw components [40]. For instance, Pradhan et al. [41] reported the development of customized 3D-printed CEs with variable pitch, established quantitative correlations between screw geometry and granule properties, and validated 3D printing for rapid prototyping of custom screw elements. However, 3D printing has been applied only to CEs, whereas customized KE designs have not yet been incorporated. In addition, the combined effects of screw element configuration and key process parameters on granule quality have not been fully explored [42].

In this study, a material–process–equipment integrated design (MPEID) methodology is proposed to address the problems mentioned above. Innovatively, the critical equipment attributes are brought forward to reflect the minimum physical attributes required from pharmaceutical unit operations. The critical equipment attributes (e.g., CSPs) are assigned equal design priority to CMAs and CPPs, forming a four-component pharmaceutical development framework that integrates CQAs with CMAs, CPPs and CSPs. By incorporating CSPs, the continuous manufacturing equipment can be modified to operate in various configurations (e.g., various screw designs in TSWG) in both development and GMP manufacturing. This advancement supports the coordinated optimization of material properties, process conditions, and equipment configurations in a holistic workflow, which constitutes the key scientific contribution of this work. The proposed MPEID methodology is applied to the continuous wet granulation process development of Guizhi Fuling capsule, which is a botanical drug [3]. The commercial production of Guizhi Fuling capsule employed high-shear wet granulation. Therefore, this study also serves as a pilot case to exemplify the transition of wet granulation of botanical formulation from batch to continuous manufacturing. Several key considerations for implementing MPEID are detailed as follows:

(1) Equivalent formulation design. For scarce and expensive raw materials, surrogate materials (e.g., pharmaceutical excipients) selected using material-similarity prediction and multivariate models are used to guide process development and reduce the experimental burden.
(2) Digital design and 3D printing of screw elements. The screw configurations are digitally characterized. Both CEs and KEs are rapidly fabricated using 3D printing.
(3) Integrated optimization of screw configuration and process parameters. The screw parameters and process parameters are systematically analyzed and integrated into a QbD-based development framework. Final validation is conducted using the actual raw materials. The CQAs of granules prepared from either batch or continuous wet granulation process are compared.

## 2. Results and Discussion

### 2.1. Equivalent Formulation Development

To identify surrogate excipients with physical properties comparable to the Guizhi Fuling compound extract powder (CEP), a PCA model (Model 1) was constructed using the data matrix described in Section 3.2 (19 physical parameters for 113 samples: one CEP sample plus 112 excipients). The first four principal components collectively explained 71.1% of the total variance, indicating that the model effectively captures the core physical property information.

PCA Model 1 was used to identify critical CMAs affecting granulation and capsule filling, including particle size distribution and powder flowability, for subsequent screening of equivalent formulations. As shown in Figure S1 of Supplementary Material S2, the first two principal components (PC1 and PC2) explained 46.7% of the total variance and captured the core variability in attributes directly linked to granulation performance: particle size distribution (*D*_10_, *D*_50_, and *D*_90_), powder flowability (*IC*, *IH*, *AOR*, and *t*), fines content (*pf%*), compressibility (*Icd*), and porosity ($\varepsilon_{p}$). In contrast, PC3 (13.7%) and PC4 (10.8%) (Figure S2 of Supplementary Material S2) primarily reflected secondary or redundant variability (e.g., particle span, true density) with weak correlation to granulation performance, as indicated by their loading patterns. Therefore, PC1 and PC2 were sufficient to capture the main variability governing granulation performance in the dataset.

**Figure 1:**
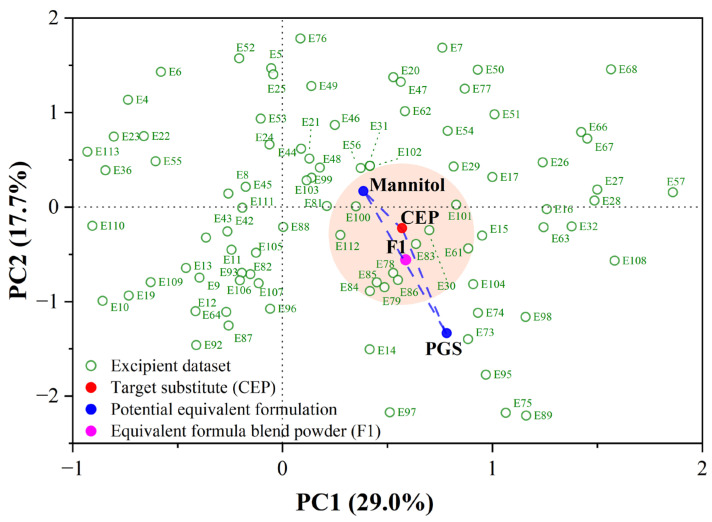
The score plot of PCA Model 1 for equivalent material screening from the excipient database. Green circles represent individual excipient candidates (labeled by code), and the purple dot denotes the optimized F1 formulation. The shaded pink area defines the region with Euclidean distance ≤ 1.0 from the CEP reference, indicating high similarity. Blue dashed lines mark the similarity boundary for the F1 formulation.

Figure 1 presents the score plot for PC1 and PC2. Red highlights indicate excipients with Euclidean distance ≤ 1.0 from CEP. Euclidean distances between each excipient and CEP (calculated in the PC1-PC2 score space) ranged from 0.05 to 8.11. Thirteen excipients with distances ≤ 1.0 were selected as candidates; among them, only dextrin (E83) and croscarmellose sodium (E30) showed high similarity (distance ≤ 0.32). Detailed material information includes the name, code, and Euclidean distances between excipient powders and CEP (Table S1 of Supplementary Material S1). Characterization data are available in Wang et al. [43].

Preliminary TSWG experiments (using 80% ethanol as the granulating liquid, consistent with the commercial CEP production process) revealed poor processability of several candidates: croscarmellose sodium and *β*-cyclodextrin swelled rapidly, leading to barrel fouling, whereas dextrins failed to form effective agglomerates (most particles < 125 μm). Consistent with Liang et al. [44], these highly hydrophilic excipients were excluded. Starch-and sugar-alcohol-based excipients were prioritized, and mannitol (E60, distance = 1.12) was selected as a primary candidate due to its favorable granulation properties (limited polymorphic transition and stable granules without additional binders) [19,22,23].

To improve similarity, a binary mixture was designed using the distance-based weighting method described in Section 3.2. Pregelatinized starch was identified as the only excipient capable of reducing the blend’s distance to CEP when mixed with mannitol. The calculations yielded the optimal formulation (No. F1): 60% (*w*/*w*) PGS and 40% (*w*/*w*) mannitol. Validation showed that the blend’s distance to CEP was 0.40, representing a >50% reduction compared with the individual excipients (mannitol: 1.12; PGS: 0.98). The blend also exhibited close clustering with CEP in the PCA score space.

**Figure 2:**
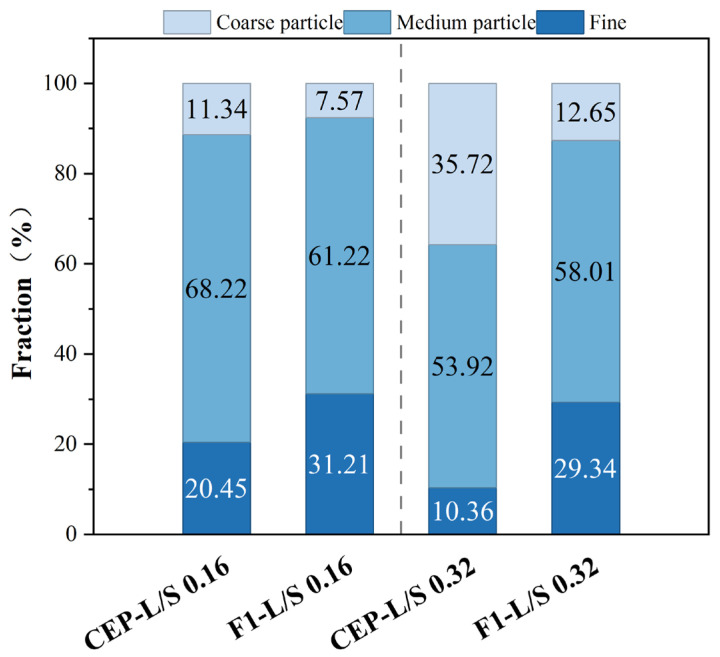
Weight-based particle size fraction distribution of TSWG granules from two formulations at different L/S ratios, determined by standard sieve analysis.

Subsequently, TSWG experiments were conducted using the CEP and the screened equivalent formulation (F1) under identical screw configuration and process conditions, with 80% ethanol used as the granulating liquid. Two L/S ratios were set for comparison: 0.16 (low) and 0.32 (high). The sieve classification results of the dried granules are presented in Figure 2.

Under the low L/S ratio, both formulations showed comparable total granule yields. At the low *L/S* ratio, the mass fraction of medium granules was 61.22% for the equivalent formulation and 68.22% for CEP, whereas the mass fraction of coarse granules was 7.57% and 11.34%, respectively. At the high L/S ratio, the discrepancies in total granule yield and fine powder content between the two formulations increased slightly, whereas their variation trends for medium granules remained highly consistent. At the high *L/S* ratio, the mass fraction of medium granules was 58.01% for the equivalent formulation and 53.92% for CEP. As the *L/S* ratio increased, the proportion of coarse granules increased markedly, accompanied by a noticeable reduction in fine powder content.

After wet granulation with 80% ethanol, the equivalent formulation achieved a stable dried granule yield of nearly 70%, comparable to CEP and within the acceptable production range. In summary, the screened equivalent formulation (F1) exhibited comparable granulation performance to CEP in TSWG and was confirmed as an alternative material for subsequent systematic continuous manufacturing research.

### 2.2. Plackett–Burman Screening Design

#### 2.2.1. ANOVA and Critical Parameter Identification for Plackett–Burman Design

The results of the PBD experiments, including medium granule yield (*Y_1_*), particle size similarity (*Y_2_*, ${\mathrm{cosine}}_{\mathrm{GSD}}$), Carr index (*Y_3_*, *IC*) and process torque (*Y_4_*), as well as the results of the analysis of variance (ANOVA), are summarized in Table S4 of Supplementary Material S1.

ANOVA was performed on particle size similarity, Carr index and process torque. For the yield of medium granules, a natural logarithmic transformation of *Y1* (*ln*(*Y1*)) was used to eliminate data skewness and stabilize variance, thereby fulfilling the normality and homoscedasticity assumptions of ANOVA. The ANOVA results showed that all main-effect models were statistically significant (*p* < 0.05): *ln*(*Y_1_*) (*p* = 0.0479), ${\mathrm{cosine}}_{\mathrm{GSD}}$ (*p* = 0.0482), *IC* (*p* = 0.0215), and *Torque* (*p* = 0.0445).

**Figure 3:**
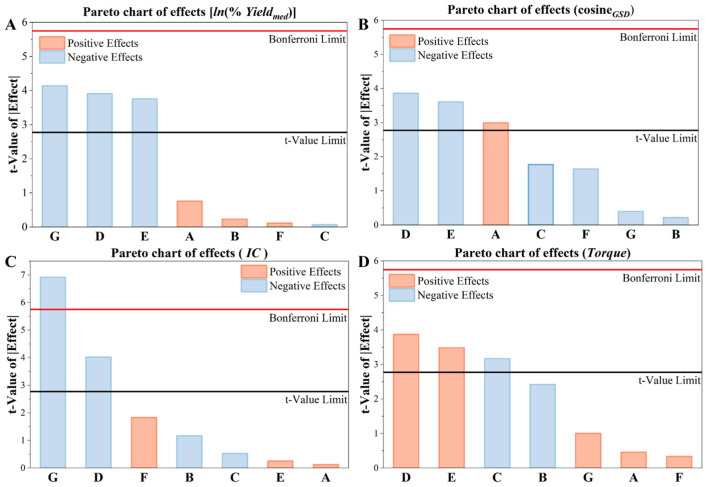
Pareto charts of response variables: (**A**) *Y_1_*, (**B**) *Y_2_*, (**C**) *Y_3_*, and (**D**) *Y_4_* in the PBD experiment. Capital letters on the horizontal axes denote the investigated TSWG parameters: A, the lead of conveying elements in the first conveying zone; B, the lead of conveying elements in the second conveying zone; C, the position of kneading elements; D, the stagger angle of kneading discs; E, the thickness of kneading discs; F, the screw speed; G, the *L/S* ratio.

The Pareto chart (Figure 3) was used to visually assess the magnitude of each factor, thereby enabling the ranking of critical parameters and clearly distinguishing positive from negative effects. Orange bars indicate factors with positive effects on the response indicators, whereas blue bars represent factors with negative effects. For the medium granule yield, the *L/S* ratio, the stagger angle and the thickness of kneading discs all showed significant negative effects. Higher values of these parameters reduced the medium granule yield. For the particle size similarity between the prepared granules and production batches, the stagger angle and the thickness of kneading discs showed significant negative effects, whereas the lead of conveying elements in the first conveying zone showed a significant positive effect. A larger stagger angle of kneading discs and a greater disc thickness lowered the particle size similarity between TSWG granules and commercial batches, whereas a longer lead of the first conveying zone conveying elements enhanced this similarity. For the Carr index, the *L/S* ratio and stagger angle of kneading discs displayed significant negative effects, with higher *L/S* and stagger angle reducing *IC* and improved flowability. For process torque, the stagger angle and the thickness of the kneading discs exhibited significant positive effects, whereas the position of kneading elements showed a significant negative effect. A larger stagger angle of the kneading discs and a greater disc thickness increased the process torque, whereas placing the kneading element farther from the liquid addition point decreased it. Larger stagger angles and thicker discs increased the material filling degree and shear resistance inside the granulator barrel, thereby directly increasing the torque required for screw rotation [45,46]. In contrast, placing the kneading element further downstream prolonged the pre-wetting and conveying time of the powder before kneading. A more sufficient and uniform liquid distribution weakened local over-agglomeration and excessive shear resistance, thereby stabilizing and lowering the process torque [17,18,47].

Overall, the ANOVA results confirmed that the critical factors affecting the TSWG of the equivalent formulation were the stagger angle and the thickness of kneading discs, the *L/S* ratio, the lead of CEs in the first conveying zone and the position of kneading elements.

To reduce the complexity and workload of subsequent BBD experiments, critical factors were selected based on Pareto chart effect magnitudes, statistical significance, and multi-indicator coverage. The stagger angle of kneading discs (factor D) showed significant effects on all four indicators (*p* = 0.0197 for *Yield_med_*; *p* = 0.0176 for ${\mathrm{cosine}}_{\mathrm{GSD}}$; *p* = 0.0158 for *IC*; *p* = 0.0171 for *Torque*) with the highest coverage and was therefore identified as the top priority. The thickness of kneading discs (factor E) significantly affected three indicators (*p* = 0.0224 for *Yield_med_*; *p* = 0.0221 for ${\mathrm{cosine}}_{\mathrm{GSD}}$; *p* = 0.0242 for *Torque*) and was recognized as the second priority. The *L/S* ratio (factor G) significantly affected two indicators (*p* = 0.0163 for *Yield_med_*; *p* = 0.0023 < 0.01 for *IC*) and was identified as the third priority. The lead of conveying elements in the first conveying zone (Factor *A*; *p* = 0.0393, ${\mathrm{cosine}}_{\mathrm{GSD}}$-only) and the position of kneading elements (Factor C) (*p* = 0.0323, *Torque*-only) significantly affected single indicators.

In summary, the critical TSWG process parameters for the equivalent formulation were ranked by statistical significance and multi-indicator coverage as follows: D > E > G > A/C. Finally, three factors were selected as the key variables for subsequent TSWG process optimization in the BBD experiments: the stagger angle of the kneading discs (D), the thickness of the kneading discs (E), and the *L/S* ratio (G).

#### 2.2.2. Non-Critical Parameter Analysis and Level Determination for Plackett–Burman Design

Non-critical parameters (A: lead of conveying elements before kneading zone, B: lead of conveying elements after kneading zone, C: kneading element position, F: screw speed) were set at predefined levels, while critical process parameters were further optimized via BBD. The levels of non-critical parameters were determined to ensure satisfactory powder flowability, particle size comparable to commercial CEP granules, high granule yield, and stable TSWG granulation performance.

As summarized in Table S4 of Supplementary Material S1, the fine powder content of PBD samples ranged from 10.08% (R8) to 41.03% (R3). The fine powder content in three experimental groups (R2, R5, and R7) was below 30%, whereas the particle yield was above 70%. The online torque ranged from 1 Nm to 12 Nm, and a higher torque indicated more intense process fluctuation and an increased risk of production during twin-screw wet granulation. Based on the torque distribution, the PBD conditions were classified into three torque levels: low (1–3 Nm), medium (4–9 Nm) and high (10–12 Nm). To maintain stable and uniform granulation, the process parameters associated with the low-torque samples (R2, R5, R7) were selected as the fixed conditions for subsequent tests.

The *D*_50_ of the PBD granule samples ranged from 13 μm to 63 μm, and the median D50 of commercial CEP industrial granules was 47 μm. Three batches (R2, R5, R7) exhibited *D*_50_ values of 43–53 μm, which were close to the industrial reference value. Their corresponding ${\mathrm{cosine}}_{\mathrm{GSD}}$ values were 0.7195, 0.7006, and 0.5303, representing the top three among all PBD groups. This result directly supports that the established ${\mathrm{cosine}}_{\mathrm{GSD}}$ indicator is accurate and reliable for quantitatively assessing the particle size similarity between TSWG granules and industrial commercial products.

*IC* ranged from 20 (R9, optimal flowability) to 32 (R8, poorest flowability). Except for sample R8, the *IC* values of the remaining 11 batches met the preliminary criteria for potential industrial production; notably, R9 and R10 exhibited the best powder flowability.

Multiple quality indicators, including particle size similarity, process torque stability, fine powder content, granule yield and flowability were comprehensively evaluated. Samples R2, R5, R9 and R12 presented low fine powder content and high granule yield. Since R2 and R5 exhibited high particle-size consistency with commercial granules and demonstrated stable low-torque performance, the parameter ranges corresponding to R2 and R5 were selected as the favorable non-critical parameter conditions for controlling particle size, fine powder content and continuous production stability. Nevertheless, R2 and R5 exhibited relatively poor flowability. To balance granule flow performance and overall quality, the non-critical parameter settings of R9 and R10, which showed superior flowability, were also considered and adopted for subsequent experiments.

The parameter configurations of the representative batches above are summarized in Table 1. Notably, R5 and R10 shared identical screw-related parameters: 36 mm for the first conveying zone, 18 mm for the second conveying zone, and a kneading element position of 22 mm. Accordingly, these screw structural parameters were fixed during the subsequent BBD optimization. The screw speed was 100 rpm for R5 and 200 rpm for R10, and marked differences were observed among the qualified batches. Previous literature has shown that screw speed exerts only a minor influence on granule properties in conventional twin-screw granulation, regardless of whether in pilot-scale or laboratory-scale equipment [48]. However, such effects may be pronounced at high channel filling ratios. Considering the overall process stability, the screw speed was finally fixed at 150 rpm, which is the midpoint of the PBD experimental range.

In summary, four screened non-critical factors were set at fixed levels for subsequent experiments. The first conveying zone used medium-lead conveying elements (MLCE, 36 mm) to ensure uniform wetting and optimal particle size similarity. The second conveying zone used small-lead conveying elements (SLCE, 18 mm) to regulate material residence time and prevent excessive agglomeration of granules. The kneading element was positioned 22 mm from the liquid addition point, enabling immediate kneading after powder wetting and improving the homogeneity of liquid distribution. A constant screw speed of 150 rpm was applied to minimize torque fluctuations and maintain stable operation.

Based on the screening results above, the unified screw configuration “MLCE-KE-SLCE-SME” was selected as the baseline setup for further BBD design. Only the geometric parameters of the kneading element were considered critical variables for subsequent optimization, whereas the remaining screw structures and non-critical process parameters were fixed at the optimal levels identified in the PBD runs.

### 2.3. Box–Behnken Response Surface Design

#### 2.3.1. ANOVA

As shown in Table S4 of Supplementary Material S1, the comprehensive evaluation results of 15 BBD experimental runs indicated that the ${\mathrm{cosine}}_{\mathrm{GSD}}$ values ranged from 0.9812 to 0.9987, the Carr index (*IC*) varied from 20.35 to 29.68, the medium granule yield was between 62.35% and 72.46%, and the torque values fell within 1.12–6.85 Nm. All response values were within the acceptable range for industrial production.

Quadratic polynomial regression models were fitted for each response variable, and the compressed regression equations, as well as ANOVA results, are summarized in Table 2. All established models exhibited excellent fitting performance with a coefficient of determination (*R*^2^ and adjusted *R*^2^) higher than 0.9, indicating a high degree of correlation between the predicted and experimental values. The predictive *R*^2^ values ranged from 0.66 to 0.79, which is acceptable for complex formulation granulation modeling. Specifically, the predictive models for *Y_1_* (*Yield_med_*) and *Y_3_* (*IC*) were constructed using a forward/backward stepwise regression approach, which automatically eliminated statistically insignificant influencing factors to obtain the optimal predictive model [49]. The predicted *R*^2^ (*R_pre_*^2^) values of all models were close to the adjusted *R*^2^ (*R_aj_*^2^) values, demonstrating good agreement between the regression models and the actual experimental data. Additionally, model assumptions were evaluated via residual analysis. As presented in Figure S3 of Supplementary Material S2, the normal probability plots of residuals showed that all data points were distributed approximately along a straight line, which verified the normality assumption. The residuals vs. predicted values plots exhibited random scattering around zero, confirming homoscedasticity. For all models, the lack-of-fit tests yielded non-significant results (*p* > 0.05) when compared with pure error. Collectively, these results ruled out underfitting and indicated that the developed models offered reliable predictive performance. The regression Equations (1)–(4) can be effectively used to predict the response values of *Y_1_* (*Yield_med_*), *Y_2_* (${\mathrm{cosine}}_{\mathrm{GSD}}$), *Y_3_* (*IC*), and *Y_4_* (*Torque*) at specific levels of the independent variables.

As illustrated in Table 2, the stagger angle of kneading discs (D) and its quadratic term (D^2^) exerted significant negative effects on both *Y_1_* (*Yield_med_*) and *Y*_2_ (${\mathrm{cosine}}_{\mathrm{GSD}}$). Similarly, the *L/S* ratio (G) and its quadratic term (G^2^) had a negative impact on *Y*_2_ (${\mathrm{cosine}}_{\mathrm{GSD}}$). The stagger angle (D) and thickness of kneading discs (E) showed negative effects on *Y*_3_ (*IC*), while the kneading disc thickness (E) and its quadratic term (E^2^) positively affected *Y*_4_ (*Torque*).

#### 2.3.2. Contour Plot Analysis and Critical Parameters Interaction Investigation

**Figure 4:**
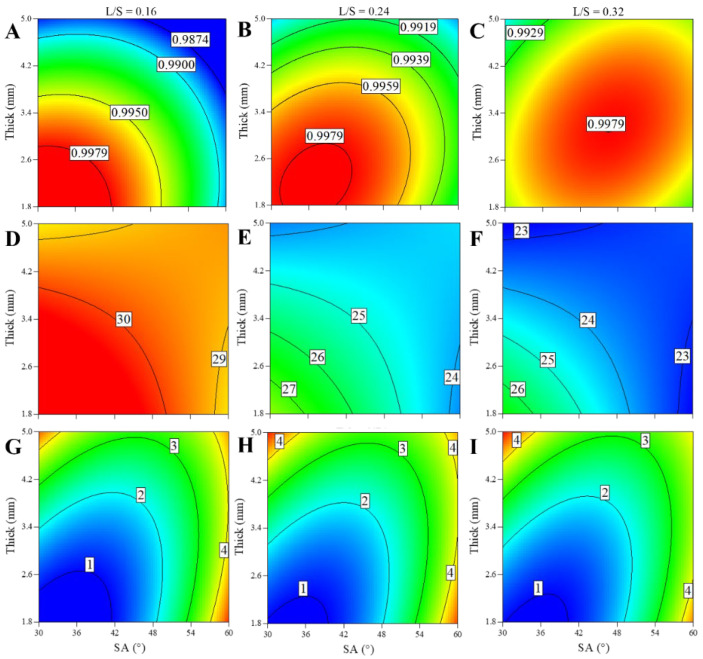
Contour plots of particle CQAs as functions of kneading element angle and kneading disc thickness (low, medium, high *L/S* ratios): (**A**–**C**) particle size similarity, (**D**–**F**) *IC* and (**G**–**I**) *Torque*. Numerical labels on the contour lines represent constant values of the corresponding response indicator. The color gradient depicts the continuous variation of the response magnitude, with blue indicating the lowest values and red indicating the highest values.

Contour plots (Figure 4) depict the interactive effects of stagger angle and the thickness of kneading discs on the particle size similarity, *IC* and Torque at three *L/S* ratios, with the third factor at the middle level. For the particle size similarity (Figure 4A–C), circular contours at low *L/S* ratio indicated weak *SA*–*thick* interaction, while elliptical contours at medium and high *L/S* revealed strong interaction. The particle size similarity decreased with increasing *SA* (30–60°) and *thick* (1.8–4.2 mm) due to insufficient liquid bridges, and the optimal particle size similarity was achieved at *SA* 30–54° and thick 1.8–3.4 mm across the tested *L/S* levels. Adequate liquid bridges supported stable interparticle bonding, and moderate shear stress induced by suitable *SA* and disc thickness promoted controlled densification and balanced granule growth. Excessively large *SA* or thick discs caused over-shear and over-densification, leading to abnormal granule growth and reduced similarity to production particles [17,18,32].

For *IC* (Figure 4D–F), the low *L/S* ratio yielded *IC* values of 29–30 (“Poor” flowability) with limited parameter interaction, while medium and high *L/S* ratios reduced *IC* values to 24–27 (“Passable”) and 23–26 (near “Fair”), respectively, with enhanced interaction and saturated flowability improvement [16]. Increased *SA* strengthened shear intensity and homogenized wet mass densification to reduce fine powder content and generate more regular granules [48], while excessive *SA* or kneading disc thickness led to irregular agglomeration. An appropriate kneading disc thickness prevented over-compaction and over-bonding to avoid a broad particle size distribution, and a higher *L/S* ratio further enhanced granule regularity and reduced interparticle friction, collectively lowering *IC* and improving flowability [18,23,28].

For *Torque* (Figure 4G–I), a distinct low-torque zone was observed at low *L/S* ratio within the *SA* range of 30–48° and *thick* range of 1.8–3.4 mm, whereas larger *SA* with thicker kneading discs increased torque values [19]. Such low-torque parameter combinations are favorable for process stability and energy saving. At the low *L/S* level, the small *SA* and thin kneading discs generated mild shear stress and moderate material filling degree, which avoided excessive friction and compaction of the insufficiently wetted powder and effectively suppressed torque fluctuation [17,18]. Overall, medium-high *L/S* (0.24–0.32) with *SA* 36–54° and *thick* 2.6–3.4 mm achieved optimal *Y_2_*, medium-high *L/S* with *SA* 45–60° & *thick* 1.8–3.4 mm (or *SA* 30–45° & *thick* 3.4–5 mm) maintained *IC* 23–25 (“Passable”), and low-torque settings guaranteed stable TSWG operation.

### 2.4. Multi-Response Optimization and TSWG Experimental Validation of Optimal Screw Configuration and Process Parameters

Multi-objective optimization was conducted in line with actual production requirements: *SA* was set as discrete values (30°, 45°, 60°) within the experimental range, while the *L/S* ratio and kneading disc thickness were continuous variables. With equal weights, the optimization aimed to maximize ${\mathrm{cosine}}_{\mathrm{GSD}}$ and medium granule yield. Under the predicted optimal screw and process parameter combination, TSWG granules were prepared and characterized in triplicate using both the equivalent formulation and CEP as per Section 3.4. Table 3 presents the optimal parameter combination (*SA* = 45°, *thick* = 2 mm, *L/S* = 0.32) and corresponding validation results. The reliability of the response surface model (RSM) was evaluated by comparing measured and predicted values and calculating the relative error (*RE*).

The comparison between measured and RSM-predicted values (Table 4) demonstrated excellent model performance in quantifying the cosine similarity of particle size distribution (${\mathrm{cosine}}_{\mathrm{GSD}}$). No significant difference was observed between the measured cosine values of TSWG granules and the predicted value (0.9970). For the equivalent formulation, measured cosine was 0.9939 ± 0.0011 (*RE* = −0.31%); for CEP, it was 0.9994 ± 0.0001 (*RE* = 0.24%). Both *RE* values were within ±1%, confirming the model’s excellent predictive performance for particle size distribution, which met production requirements.

Process optimization significantly improved particle flowability (*IC*) and process stability. *IC* decreased from 26.67 (“Passable Flowability”) for the equivalent formulation to 20.39 (“Fair Flowability”) for CEP. The standard deviation of CEP *IC* (±0.31%) was 67% lower than that of the equivalent formulation (±1.05%), ensuring better stability for continuous production. However, *IC* and torque prediction accuracy showed significant formulation dependence. For the equivalent formulation, measured *IC* was 26.67 ± 1.05 (*RE* = 7.49%), within the acceptable range (*RE* ≤ 10%). In contrast, CEP’s measured *IC* (20.39 ± 0.31) was significantly lower than the predicted value (24.67, *RE* = −20.97%) and equivalent formulation (6.28 percentage points lower), as well as the average production granule *IC* (23.89%, 3.5 percentage points lower), but still within production *IC* fluctuation range. This negative error (*RE* = −20.97%) indicated superior actual flowability of the CEP validation batch, highlighting successful process optimization. The achieved *IC* was consistent with “Excellent” flowability as defined by the Jenike standards, accompanied by an approximate 8° reduction in the angle of repose. This improvement may enhance the filling qualification rate of CEP capsules.

CEP torque prediction was reliable (measured: 1.48 Nm, predicted: 1.38 Nm, *RE* = 6.62%) and expected to be more energy-efficient than the conventional production torque (1.95 Nm). However, the equivalent formulation torque showed a significant positive deviation (measured: 1.94 ± 0.0 Nm, predicted: 1.38 Nm, *RE* = 28.76%). Although the measured torque was far below the equipment shutdown threshold (20 Nm), indicating potential for TSWG process preliminary screening, its significant systematic deviation requires caution. Differences in torque across formulations are attributed to the variations in powder density. Under identical processing conditions, the F1 formulation exhibited higher torque than the CEP formulation owing to its greater bulk density, which increased the filling degree of the granulator barrel. Furthermore, the elevated filling degree amplified the swelling behavior of pregelatinized starch and the wetting properties of mannitol (a water-soluble filler). These factors collectively increased frictional resistance, leading to a torque prediction relative error of 28.76%. No processability issues were detected, as both formulations maintained relatively low torque values during granulation [10]. The model developed from static powder properties is inherently limited when predicting dynamic torque, yet it provides reliable results for core granule quality attributes. It should be noted that this model is not applicable for high-precision torque prediction and on-site process control, and potential deviations in parameter settings require careful attention, but it conducts preliminary processability for TSWG process conditions. Under the present laboratory-scale conditions, this formulation is unsuitable for accurate torque prediction and process control, and the associated parameter setting deviation risks should be noted.

### 2.5. PCA Biplot Analysis for Process Transferability Verification and Multi-Batch Quality Consistency Evaluation

To further evaluate the property characteristics of TSWG process validation samples, verify the predictability of the response surface model, and confirm the stability and transferability of the BBD-optimized process, a PCA model was established using a 126 × 5 property matrix. This matrix included five key parameters (*D*_10_, *D*_50_, *D*_90_, *Span*, and *IC*) from 126 samples. The PCA Model 2 was constructed with the first three principal components. A 7-fold cross-validation was applied to assess model robustness, with cumulative *R_X_*^2^ = 0.94.7 (PC1: 51.5%, PC2: 30.1%, PC3: 13.1%) and *Q*^2^ = 0.720, indicating good explanatory and predictive capacity of the PCA Model 2.

**Figure 5:**
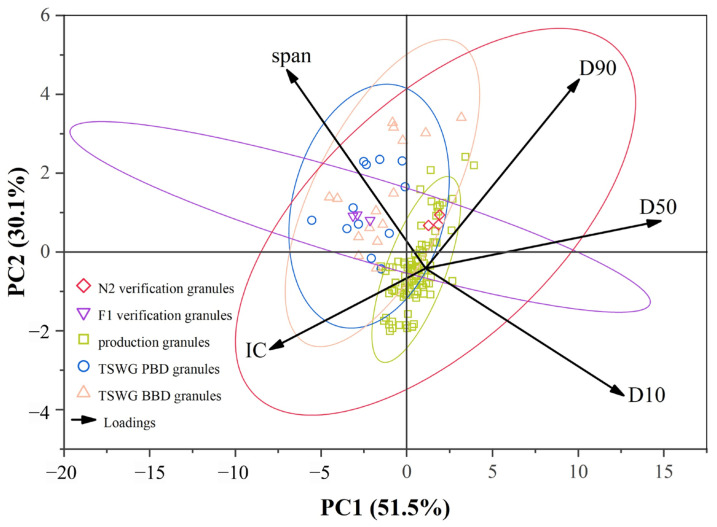
The PCA biplot (Model 2) for process transferability and quality consistency verification. The plot displays score distributions of five granule sample groups (with 95% confidence interval ellipses, colored by group: green for commercial production granules, blue for TSWG PBD equivalent formula granules, orange for TSWG BBD equivalent formula granules, purple for F1 validation granules, red for TSWG CEP validation granules) and loading vectors of key particle size/flowability parameters.

Figure 5 shows the biplot (superimposed score and loading plots) of the first two principal components, with 95% confidence intervals (ellipses) for each sample type (different colors represent different sample groups: green for 93 batches of commercial CEP production granules, blue for 12 batches of TSWG PBD equivalent formula granules, orange for 15 batches of TSWG BBD equivalent formula granules, purple for three batches of TSWG equivalent validation granules (No. F1), and red for three batches of TSWG CEP validation granules (No. N2).

Loading vectors in the biplot indicate each parameter’s contribution to principal components. PC1 was dominated by positive *D*_10_, *D*_50_, and *D*_90_ loads (load > 0.8), quantifying particle size differences. Production granules had significantly higher PC1 scores than PB blank granules, consistent with their higher average *D*_50_ (57.89 μm vs. 37.49 μm). PC2 (“particle size distribution-flowability balance factor”) was regulated by positive *Span*, *D*_90_ loads and negative *IC* load (load ±0.7). Production granules showed mainly negative PC2 distribution (*IC*: 15.85–28.65), reflecting high flowability fluctuation; TSWG blank granules showed positive PC2 shift, indicating wide distribution and high flowability. BBD-optimized samples had PC1 scores close to production granules and positive PC2 migration, confirming synergistic particle size and flowability improvement via process parameters.

All CEP validation granules (*n* = 3) fell within the 95% confidence interval of 93 production batches (average *D*_50_: 58.3 ± 11.1 μm, average *IC*: 23.9 ± 2.3%), meeting production requirements. Meanwhile, for CEP validation granules, *D*_10_ and *IC* showed CV values below 3% (2.22% and 1.52%, respectively), while CV values of *D*_50_, *D*_90_ and *Span* ranged from 3.83% to 8.68%. All parameters presented far smaller variation than commercial production samples. The blank validation granules (*n* = 3) aggregated in the PB/BBD blank confidence interval but showed significant PC1 negative deviation (*D*_50_ reduced by 6.8 μm), revealing the model’s limitation of excluding formulation factors. TSWG validation granules concentrated in the PC1 negative quadrant (*D*_90_ reduced by >15%) with near-zero PC2, confirming large particle inhibition and flowability optimization.

The PCA biplot enables rapid new batch compliance assessment, reducing particle size and flowability detection frequency and providing a preliminary basis for potential industrial continuous production quality control. PCA results confirm that CEP validation granules meet production specifications, and the optimal process improves blank granule properties to approach production batches, verifying TSWG process repeatability and scale-up transfer potential.

### 2.6. Capsule Filling Weight Variation Evaluation and Comparison Between Equivalent Formulation and CEP TSWG Validation Granules

According to Chinese Pharmacopoeia 2025 [50], the content uniformity test is only mandatory for hard capsules with a labeled weight less than 25 mg or formulations where the main active ingredients account for less than 25% of the unit weight. The Guizhi Fuling capsules used in this study have a labeled weight of 0.31 g (310 mg), so content uniformity testing is not required. The weight variation test is adopted as the official alternative test to evaluate the uniformity of capsule fill weight.

**Figure 6:**
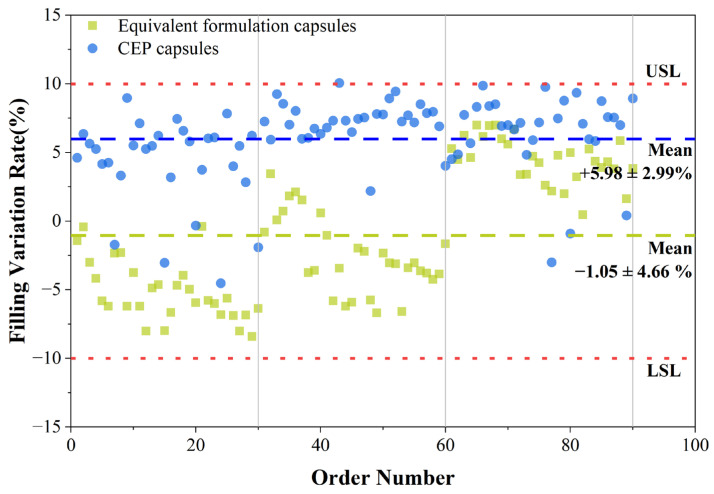
Filling variation diagram of capsules filled with validation experimental granules Blue circles represent CEP capsules, and yellow squares represent equivalent formulation capsules. Red dashed lines indicate the upper specification limit (USL, +10%) and lower specification limit (LSL, −10%) defined by the Chinese Pharmacopoeia 2025. The blue dashed line denotes the mean filling variation rate of CEP capsules (+5.98 ± 2.99%), while the green dashed line denotes the mean filling variation rate of equivalent formulation capsules (−1.05 ± 4.66%).

Figure 6 shows capsule filling weight variation (*n* = 90), with red dotted lines indicating the lower specification limit (LSL: 0.2790 g) and the upper specification limit (USL: 0.3410 g) defined in the Chinese Pharmacopoeia (ChP) 2025 Edition. The blue and green dashed lines represent the mean filling weight variation of CEP capsules and equivalent formulation capsules, respectively. For each formulation, three parallel validation tests were conducted (Section 3.5.2), with three random samplings (10 capsules each) totaling 90 capsules per formulation. Filling variation rate was calculated as the percentage of the difference between single capsule weight and labeled weight (0.31 g) relative to the labeled weight. All batches met the Pharmacopoeia requirements, with no unqualified samples.

Under identical filling parameters, CEP samples had a higher filling weight range (0.2960–0.3412 g, average 0.3285 g) than the equivalent formulation (0.2840–0.3317 g, average 0.3068 g), a 21.7 mg difference. This was directly related to physical properties: the equivalent formulation’s moderate flowability (*IC* = 26.67), high fine particle proportion, and wide distribution (*Span* > 3.0) caused insufficient filling uniformity, with some weights approaching LSL (within 1.8% of the LSL). In contrast, CEP’s excellent flowability (*IC* = 20.39) and uniform large particle distribution ensured sufficient filling, with some weights approaching USL (0.06% from the USL).

Descriptive and inferential statistical analyses were performed for the filling variation rate data. The filling variation rate was −1.05 ± 4.66% (median: −2.24%; interquartile range: 8.90%) for the equivalent formulation, and 5.98 ± 2.99% (median: 6.77%; interquartile range: 2.42%) for CEP. Shapiro–Wilk normality tests confirmed both datasets were non-normally distributed (*p* < 0.05), and the non-parametric Mann–Whitney U test revealed a significant difference between the two groups (*p* < 0.05). The CEP group exhibited a markedly narrower interquartile range and more concentrated data distribution, indicating significantly better filling uniformity and operational stability. Notably, the slight over-limit risk of CEP was a benign outcome attributed to its unexpectedly superior granule flowability, which led to higher filling density under identical parameters. Such excellent flowability is conducive to increasing capsule filling speed and improving production efficiency in future continuous production processes.

This confirms that the optimal TSWG process achieves qualified filling weights for both formulations via particle flowability and size distribution regulation. To optimize filling distribution and reduce deviation, reduce filling rod speed (current 15 mm/s) for the equivalent formulation to mitigate fine powder stratification, and improve level sensor sensitivity (current threshold ± 2%) for CEP to inhibit overfilling.

### 2.7. Dissolution Profile Comparison and Consistency Evaluation Between TSWG-Validated CEP Capsules and Commercial Capsules

**Figure 7:**
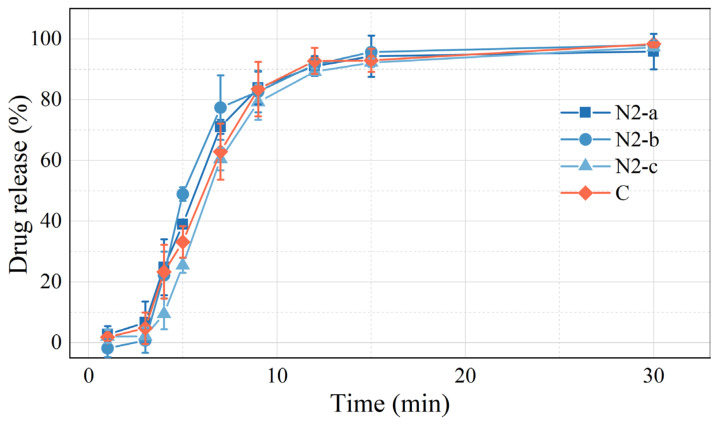
Cumulative dissolution and release rate curves of validation experimental CEP capsules and commercial capsules.

As shown in Figure 7, CEP validation capsules (N2-a, N2-b, and N2-c; blue curve) disintegrated and dissolved at 3–5 min (average 4 min), with complete capsule shell dissolution at 7–9 min. At 15 min (five minutes after shell dissolution), the average cumulative dissolution rates of three parallel replicates were 94.4%, 95.7%, and 92.2%, consistent with Zhao et al. [51], confirming rapid dissolution. Commercial CEP capsules (No. C; red curve) started dissolving at 3–4 min (more samples initiated dissolution at 4 min), with concentrated dissolution between 4 and 12 min and subsequent plateauing of the curves.

For immediate-release oral solid preparations, dissolution profiles can be judged as comparable when the cumulative dissolution percentage of both test and reference products reaches ≥ 85% within 15 min, and similarity factor (*f*_2_) analysis is not necessary under this condition. In this study, the 15 min cumulative dissolution rate of TSWG CEP validation capsules exceeded 90%, confirming they had similar dissolution behavior to commercial capsules and meeting existing quality control requirements.

These findings further verified the feasibility and stability of the TSWG granule drug release performance. Specifically, the Guizhi Fuling capsules produced under the optimized TSWG process exhibited rapid dissolution properties that meet the production requirements of immediate-release preparations, which also provides preliminary support and potential industrial application of continuous TSWG technology in botanical drug oral solid formulations.

## 3. Materials and Methods

### 3.1. Materials

Guizhi Fuling capsules (GFCs) and their corresponding composite extract powder (CEP) were provided by Jiangsu Kanion Pharmaceutical Co., Ltd (Lianyungang, Jiangsu, China). The composition of GFCs and the properties of CEP have been described in detail in our previous work [3]. The two-excipient blended formula (F1) contained 60% (*w*/*w*) pregelatinized starch (PGS, Batch No.: 2024121301, Shandong Liujia Pharmaceutical Excipient Co., Ltd., Zibo, Shandong, China) and 40% (*w*/*w*) mannitol (XL Opal, Batch No.: 122000024, SPI Pharma, Inc., Wilmington, DE, USA). Information for other excipients is listed in Table S1 of Supplementary Material S1. All physical property data were determined using consistent methodologies, with excipient data obtained from the public iTCM material database established by our research group.

An 80% (*v*/*v*) ethanol solution (Fuchen Chemical Reagent Co., Ltd., Tianjin, China) was used as the granulation liquid. Black Resin V5.0 (Form 4, Formlabs, Somerville, MA, USA) was used as the photopolymer resin for 3D-printed screw elements.

### 3.2. Construction of Powder Physical Property Database and Equivalent Formulation Development

Powder characterization was performed according to the material characterization protocol established in the iTCM database [52]. Nineteen physical parameters were determined, covering six major attributes: basic properties, particle size, compressibility, flowability, stability, and homogeneity. Corresponding abbreviations and units are summarized in Table 5 [47].

Given the limited availability of the CEP raw material, large-scale experimentation was not feasible. To reduce development costs for TSWG, excipients or formulations with comparable physicochemical properties were screened as suitable surrogates for CEP. CEP was fully characterized using the same iTCM method to establish a target property profile. Property data for 112 excipients were retrieved from the iTCM database, all measured under identical conditions.

PCA Model 1 was constructed based on a data matrix comprising 19 physical parameters across 113 samples (CEP plus 112 excipients). The Euclidean distances ($d_{s}$) between each excipient and CEP were calculated in the two-dimensional score space defined by the first two principal components (Equation (5)). A smaller $d_{s}$ indicates higher similarity in physical properties. The Euclidean distance results are listed in Table S1 of Supplementary Material S1.

(5) $$d_{s}=\sqrt{\sum (x_{s}-x_{CEP}{)}^{2}+{\left(y_{s}-y_{CEP}\right)}^{2}}$$

where $d_{s}$ represents the Euclidean distance between the *s*-th material and CEP, x is the score of the sample on PC1 of PCA Model 1, and y is the score on PC2.

Based on Euclidean distances, the mass fraction of each candidate excipient was determined using a distance-based weighting approach (Equation (6)):

(6) $$R_{s}\%=\frac{d_{s}}{\sum_{s=1}^{N}d_{s}}\times 100$$

where *N* is the number of components in the formulation, and *R*_s_% represents the calculated proportional percentage of the *s*-th component.

To strengthen the validation of the PCA-derived equivalent formulation strategy beyond physical property matching, TSWG trials were implemented for CEP and the screened equivalent formulation using unified screw geometries and process parameters (Section 3.4.3). 80% ethanol, the conventional granulating solvent for industrial batch production of CEP, was employed in all tests. The *L/S* ratios were investigated to define the practical *L/S* range. Post-granulation sieve analysis was conducted to characterize particle size distribution and assess the similarity in granulation behavior.

### 3.3. Digital Design and 3D Printing of Customized Screw Elements

**Figure 8:**
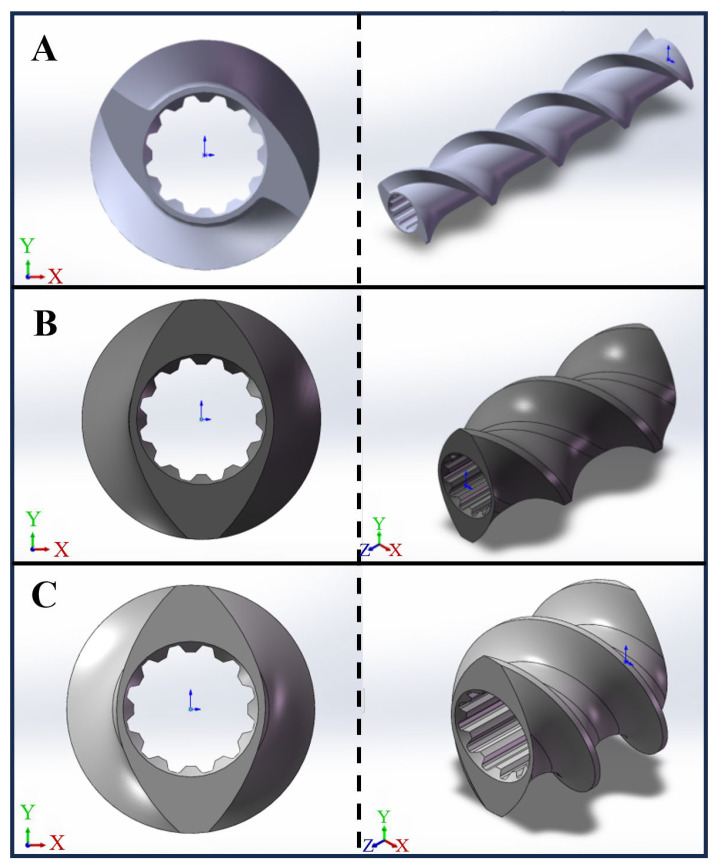
Conveying elements used in this study: (**A**) LLCE with lead 45 mm and length 5D; (**B**) MLCE with lead 36 mm and length 2D; (**C**) SLCE with lead 18 mm and length 1D.

The geometric design of all screw elements includes conveying elements and kneading elements. Geometric parameters for conveying elements included lead and length-to-diameter ratio (L/D, D = 18 mm). Based on flight lead, three types were defined: small (SLCE, 18 mm; Figure 8A), medium (MLCE, 36 mm; Figure 8B), and long (LLCE, 45 mm; Figure 8C). Conveying elements with six different L/D ratios (L/D: 0.5, 1, 1.5, 2, 4, and 5) were assembled as required.

**Figure 9:**
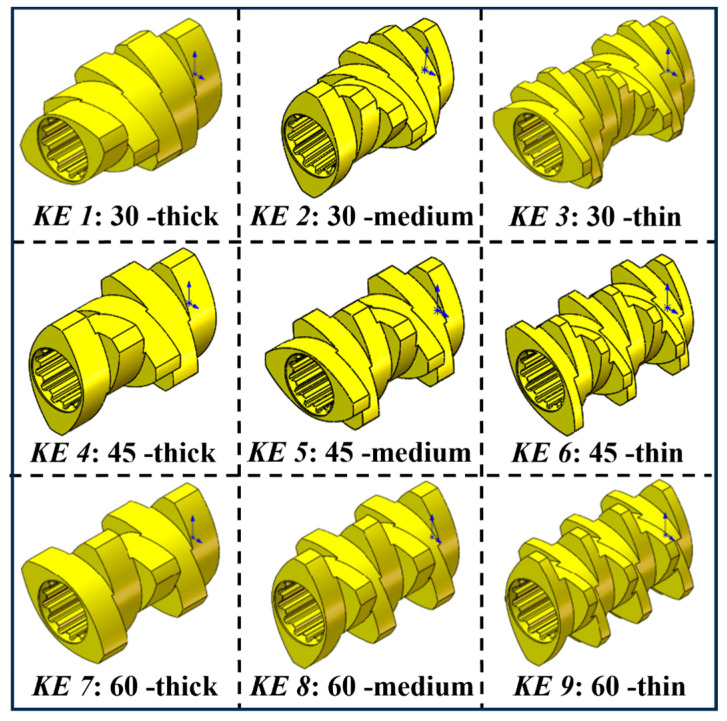
Kneading elements with *SA* values of 30°, 45°, and 60° and disc thicknesses of 1.8, 3.4, and 5.0 mm.

Kneading elements were characterized by kneading disc thickness and stagger angle (*SA*), with a fixed element length of 1.5D in this study. Three *SA* levels (30°, 45°, 60°) and three disc thicknesses (thin: 1.8 mm; medium: 3.4 mm; thick: 5 mm) were investigated. A clearance of 0.5~1 mm between adjacent discs was maintained to prevent abrasion, resulting in 5~10 discs per kneading element. According to the experimental design in Section 3.4.2, nine kneading element configurations were generated and coded as follows: 30-thick (*KE 1*), 30-medium (*KE 2*), 30-thin (*KE 3*), 45-thick (*KE 4*), 45-medium (*KE 5*), 45-thin (*KE 6*), 60-thick (*KE 7*), 60-medium (*KE 8*), and 60-thin (*KE 9*), as illustrated in Figure 9.

Geometric models of conveying elements and the 30-medium kneading element were provided by Pharmaceutical Processing Solution Co., Ltd. (PPS, Suzhou, China), as these elements were employed in the PPS TSE18 twin-screw granulator. All other screw elements were designed using SolidWorks 2021 software (Dassault Systèmes, Vélizy-Villacoublay, France) and 3D-printed with Black Resin V5.0 (Form 4, Formlabs, Somerville, MA, USA) on a Decisive Tegu Form 4 printer (Formlabs, Somerville, MA, USA) to ensure satisfactory meshing. The Form 4 printer (50 μm pixel size) provides dimensional accuracy of ±0.15% (±0.02 mm lower limit) for 1–30 mm screw features, meeting twin-screw meshing requirements. The printed elements had smooth surfaces without visible layer lines, compatible with twin-screw granulation requirements, and maintained consistent quality supported by the printer’s high process reliability.

### 3.4. Particle Preparation and Physical Characterization

#### 3.4.1. Plackett–Burman Design for Critical Parameter Screening

A Plackett–Burman design was employed to systematically evaluate the effects of screw configuration and process parameters on TSWG granule quality attributes and to screen out the critical parameters with significant impacts for subsequent optimization. The PBD is an efficient two-level screening method with low (−1) and high (+1) levels, which allows identification of significant main effects using a minimal number of runs via fractional factorial design (*n* = multiple of *k* + 1, where *k* is the number of factors), making it suitable for rapid screening of key variables in multi-factor systems. It was worth noting that the PBD inherently exhibited confounding between main effects and low-order interaction effects. Since the PBD at this stage was merely applied to screen factors with significant main effects instead of evaluating interaction effects, this confounding did not affect the reliability of factor screening results.

Factors and levels used in the PBD are listed in Table 6, with ranges determined from preliminary experiments. Seven independent variables were investigated: lead of CEs in the first conveying zone (A), lead of CEs in the second conveying zone (B), position of the kneading element (C), stagger angle of the kneading discs (D), thickness of the kneading discs (E), screw speed (F), and *L/S* ratio (G).

Each factor was set at two fixed levels (low level: −1, high level: +1), with specific settings as follows: the thread lead of CEs in the first conveying zone (the region before the kneading zone) was set to 36 mm and 45 mm, as multiples of the screw diameter (18 mm) to ensure stable conveyance, and that of the second conveying zone (the region after the kneading zone) was set to 18 mm and 36 mm; the position of the kneading element, defined as the axial distance from the liquid addition point to the start of the kneading element, was set to a low level of 22 mm and a high level of 175 mm; for the single kneading zone with fixed total length of 27 mm (adopted because preliminary tests showed poor dissolution of CEP capsules with two kneading zones), the kneading discs thickness was set to 1.8 mm and 5 mm based on commercial screw element specifications, with a 0.5–1 mm gap reserved between adjacent discs to prevent mechanical abrasion during operation [53], and the stagger angle set to 30°and 60° because preliminary experiments and literature confirmed 90° induced excessive torque (>20 Nm) and process shutdown; and screw speed was set to 100 rpm and 200 rpm, and the *L/S* ratio was set to 0.16 and 0.32 as determined by the standard “fist test” [47] to ensure adequate wetting and granulation. A global constraint was applied that the thread lead of CEs in the first conveying zone was no lower than that of the second zone in all runs, which was compatible with the granulator’s commercially available conveying elements.

Four responses were evaluated: yield of the medium particle fraction determined via sieve analysis (*Y_1_*: *Yield_med_*), cosine similarity of granule size distribution (*Y_2_*: ${\mathrm{cosine}}_{\mathrm{GSD}}$) between trial and production granules (calculated based on the *D*_10_, *D*_50_, *D*_90_, and *Span* values), Carr index (*Y_3_*: *IC*), and average torque at steady-state granulation (*Y_4_*: Torque). A two-level PBD involving seven factors was designed using Design-Expert 13.0 (Stat-Ease, Minneapolis, MN, USA), generating 12 experimental runs (Table S2 of Supplementary Material S1). Significant factors identified from the PBD were further optimized using a Box–Behnken design (BBD).

#### 3.4.2. Box–Behnken Design for Critical Process Optimization

A Box–Behnken design was adopted to further optimize the critical screw configuration and process parameters screened out via the previous PBD. As a classical second-order response surface design, BBD can quantitatively evaluate main effects, two-factor interaction effects and quadratic effects simultaneously, and it can construct a reliable regression model for process prediction and optimization. In addition, BBD does not contain experimental points at the extreme levels of factors, making it more suitable and stable for pharmaceutical granulation operations. Three critical factors were set at three levels (−1, 0, +1), with detailed parameter settings shown in Table 7. Other non-critical parameters were fixed at the optimal levels determined in Section 2.2.2. The BBD design included 3 replicates at the center point, with a total of 15 experimental runs. The full design matrix and corresponding experimental results are listed in Table S3 of Supplementary Material S1. All experimental data were fitted to a second-order polynomial regression model to correlate the granule quality responses with the factors, with the significance level set at *p* < 0.05. Data analysis was performed using Design-Expert 13 software.

Multi-response optimization was performed to simultaneously maximize the yield of medium granules (*Y_1_*) and the cosine similarity of particle size distribution (*Y_2_*) (the two core response indicators of the established regression models), with equal weighting set for both targets. The desirability function method, the mainstream multi-response optimization approach for response surface methodology, was adopted for calculation. During optimization, the kneading disc stagger angle was set as a discrete variable (fixed optional values: 30°, 45°, 60°), while the *L/S* ratio and kneading disc thickness were set as continuous variables. The optimal screw configuration and process parameters were predicted via the regression model, and triplicate validation experiments were subsequently conducted under the optimal conditions to confirm that the granule quality met industrial production requirements.

#### 3.4.3. Twin-Screw Wet Granulation and Drying

**Figure 10:**
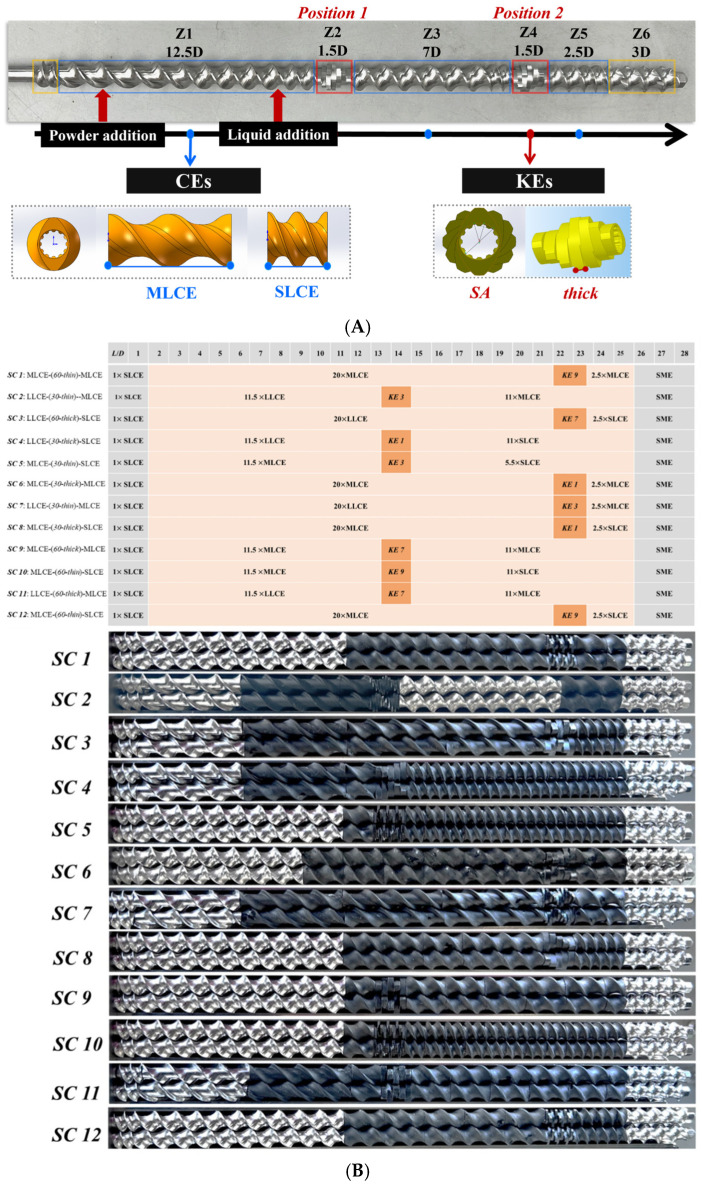
(**A**) Schematic diagram of twin-screw granulator. The black arrow indicates the direction of material flow. The upstream yellow box represents the fixed conveying element, and the downstream yellow box represents the fixed shearing zone. Blue boxes denote adjustable conveying zones, and red boxes represent kneading zones at two different axial position levels; both are the screw design regions investigated in this study. Red arrows indicate the powder and liquid addition ports, respectively; (**B**) PB experiment screw configurations.

All TSWG experiments were performed using a PPS TSE18P twin-screw granulator. The screws possessed a diameter of 18 mm and an L/D ratio of 28:1. Liquid and powder feed ports were located at 210 mm and 30 mm from the granulator inlet, respectively (Figure 10A). Modular and interchangeable screw elements were mounted on shafts to construct various screw configurations. Conveying, kneading, and shearing elements were adopted as the main element types, which formed conveying (blue-lined area in Figure 10A), kneading (red-lined area), and shearing zones along the screw shaft. All configurations employed in this study were equipped with a 1D conveying element with a lead of 18 mm at the upstream section to fix the relative position of the twin screws and prevent backflow. A reverse shallow-paddle mixing element (SME) with a length of 3D was consistently installed at the discharge end to establish a shearing zone and reduce the formation of oversized agglomerates (yellow-lined area in Figure 10A). The remaining screw length (24D) was defined as the DoE zone, where screw elements were arranged according to the DoE matrices shown in Tables S2 and S3 of Supplementary Material S1.

During the equivalent material screening stage, a fixed screw configuration was used, as presented in Figure 10A. It contained two kneading zones, each consisting of one “30-medium” kneading element. Before the first kneading block, one LLCE (5D), two MLCEs (1D, 4D), and one conveying element with a lead of 27 mm (1.5D) were arranged sequentially. The two kneading blocks were separated by two MLCEs (4D, 2D) and one SLCE (1D). After the second kneading block, one conveying element with a lead of 27 mm (1.5D) and one SLCE (1D) were incorporated. For subsequent experimental stages, different screw configurations were applied according to the DoE scheme. Twelve configurations (*SC 1*–*SC 12*) were used in the Plackett–Burman screening stage (Figure 10B). Configurations adopted in the Box–Behnken optimization stage maintained identical conveying zones to *SC 5* in Figure 10B and differed only in the kneading elements used (Figure 9).

After thoroughly mixing the pregelatinized starch and mannitol in the specified proportions, the mixture was fed into the integrated PPS F18 volumetric feeder of the granulator. Binder liquid was delivered to the liquid nozzle via a calibrated peristaltic pump (LabN3-III, Innofluid Co., Ltd., Shanghai, China). Premixed powder was wetted at the liquid addition point within the first conveying zone. The wet mass was intensively mixed by the downstream kneading blocks and conveying elements and finally discharged after passing through the terminal shearing zone. The barrel jacket was preconditioned and maintained at 20 °C throughout all experiments. Screw speed and the *L/S* ratio were set according to the experimental design. The *L/S* ratio was regulated by adjusting the mass flow rates of powder and liquid. The feeder speed was set between 15 and 30 rpm, corresponding to a screw speed range of 100–200 rpm and a throughput of 2–6 kg/h, to maintain a consistent fill level in the barrel. Prior to each run, the built-in torque sensor was zero-calibrated under idle conditions. Torque data were continuously acquired at a sampling frequency of 1 Hz throughout granulation, and real-time signal monitoring was conducted to exclude signal drift and ensure reliable measurement. The system reached a steady state with constant torque after approximately 5 min of operation. Only torque data collected over a continuous 60 s stable interval under steady state were used for analysis. Around 600 g of wet granules were then sampled from the discharge port.

Wet granules were sieved through a standard 24-mesh (850 μm) screen for wet sizing, uniformly distributed on trays, and dried in a thermostatic drying oven (DHG-9030, Shanghai Hengyi Scientific Instrument Co., Ltd., Shanghai, China) at 45 °C for 24 h until the residual moisture content was below 5%. Moisture content was determined using a rapid moisture analyzer (ML-03F, Shenzhen Guanya Instrument Technology Co., Ltd., Shenzhen, China) equipped with an infrared dryer and analytical balance. Granule samples (2 g) were dried at 105 °C to constant weight within 30 s. Dried granules were resieved through a standard 24-mesh screen for dry sizing and stored for subsequent characterization of particle size, yield, and flowability.

#### 3.4.4. Particle Size Fractionation by Sieving

Dried granules were sieved with an analytical sieve shaker (ANS-300, Beijing Xingshi Lihe Technology Development Co., Ltd., Beijing, China). A stacked set of standard test sieves with aperture sizes of 850 μm, 355 μm, 250 μm and 125 μm was adopted for granule classification. The sieving operation was carried out at an amplitude of 1.5 mm for 5 min. The retained materials on each sieve were collected and weighed separately, and the mass fraction of each granule fraction was calculated according to Equation (7).

The total granule yield was defined as the mass proportion of particles in the range of 125–850 μm. Particles smaller than 125 μm were defined as fine powders. The qualified yield fraction was further divided into coarse granules (355–850 μm, *Yield_coarse_*) and medium granules (125–355 μm, *Yield_med_*, *Y_1_*). All classified granule fractions were collected and stored for subsequent particle size characterization.

(7) $$w_{i}(\%)=\frac{m_{i}}{\sum m_{i}}\times 100$$

*w_i_* is the mass fraction of each separated granule fraction, and *m_i_* is the mass of the corresponding sieve fraction sample.

#### 3.4.5. Particle Size Analysis and Similarity Calculation

Granule size distribution (GSD) was determined via the laser diffraction method. Granule samples from Plackett–Burman design runs were measured using a BT-9300LD laser particle size analyzer (Dandong Baite Instruments Co., Ltd., Dandong, Liaoning, China). All remaining samples were characterized with a Mastersizer 2000 (Malvern Panalytical, Malvern, Worcestershire, UK) coupled with a Scirocco 2000 dry dispersion module. GSD data were calculated from light scattering signals based on the Mie scattering model. Approximately 1 g of each sample was tested, and all measurements were performed in triplicate to ensure reproducibility.

Given the typical broad and bimodal distribution of TSWG granules, direct one-time detection may cause signal interference and algorithm deviation at the particle size boundary. To improve testing accuracy, a sieving-segmented detection and linear summation method was established. Granule samples were divided into four independent size fractions in accordance with the classification method described in Section 3.4.4, and each fraction was determined individually. Comprehensive GSD indicators, including *D*_10_, *D*_50_, *D*_90_, and *Span*, were obtained by linear summation calculation (Equation (8)) following the ideal mixing rule [47].

GSD cosine similarity (${\mathrm{cosine}}_{\mathrm{GSD}}$, *Y_2_*) was introduced to quantitatively evaluate the consistency between prepared granules and the average data of 97 commercial production batches. Four core GSD parameters (*D*_10_, *D*_50_, *D*_90_, and *Span*) were selected as evaluation indicators. Before calculation, z-score normalization was performed in MATLAB R2023a (The MathWorks Inc., Natick, MA, USA) to eliminate dimensional differences among multiple variables. The GSD cosine similarity was calculated according to Equation (9).

(8) $$P_{1k}=\sum (w_{i}\times P_{1ki})$$

where *P*_1*k*_ represents the standardized value of the *k*-th GSD parameter of the test sample, and *P*_1*ki*_ is the particle size parameter of the independent sieve fraction.

(9) $${\mathrm{cosine}}_{\mathrm{GSD}}(\theta )=\frac{\sum_{k=1}^{n}P_{1k}P_{2k}}{\sqrt{\sum_{k=1}^{n}{P_{1k}}^{2}}\sqrt{\sum_{k=1}^{n}{P_{2k}}^{2}}}$$

where ${\mathrm{cosine}}_{\mathrm{GSD}}(\theta )$ represents the vector cosine similarity between two groups of multi-dimensional particle size data, ranging from 0 to 1 (a value of 1 indicates complete consistency). The dimension number *n* was set as 4, corresponding to the four GSD parameters: *D*_10_, *D*_50_, *D*_90_, and *Span*; *P_1k_* represents the standardized value of the *k*-th GSD parameter for the test granule sample; and *P_2k_* represents the standardized mean value of the *k*-th GSD parameter for the 97 production batches.

#### 3.4.6. Bulk Density, Tapped Density and Carr Index

Bulk density (*D*_a_) and tapped density (*D*_c_) were measured to calculate the Carr index (*IC*, *Y_3_*), an indicator of granule flowability:

(10) $$IC=(1-\frac{D_{a}}{D_{c}})\times 100$$

where *IC* is the Carr index, *D*_a_ is the bulk density (g/mL), and *D*_c_ is the tapped density (g/mL).

### 3.5. Capsule Preparation and Quality Assessment

#### 3.5.1. Capsule Preparation

Granules from validation experiments were filled into size 0 pharmaceutical hollow capsules using an NJP-400A automatic capsule filling machine (Tianjin Hanlin Hangyu Industry Co., Ltd., Tianjin, China). The target filling weight was controlled at 310 mg (weight deviation: ±10%) by adjusting the level sensor and filling rod depth. Filled capsules were visually inspected to exclude damaged or powder-leaking samples prior to subsequent quality evaluation.

#### 3.5.2. Capsule Weight Variation

Weight variation was inspected in accordance with General Chapter 0103 “Capsules” of the ChP 2025 Edition, Volume IV [50].

Ten capsules from each validation batch were randomly selected and individually weighed using an electronic analytical balance (GL124-1SCN, Sartorius AG, Göttingen, Germany). Each capsule was carefully opened, the shell was weighed separately, and the net content weight was calculated as the difference between total weight and shell weight. The individual weight variation rate was determined using Equation (11).

Acceptance criteria: For labeled weights of 0.3 g, the weight variation limit was ±10%. No more than 2 capsules per batch were allowed to exceed this limit, and no capsule exceeded twice the limit.

(11) $$\mathrm{weight}\;\mathrm{variation}\;(WV\%)=\frac{W_{i}-W_{s}}{W_{s}}\times 100$$

where *W_i_* is the content weight of an individual capsule (g), and *W_s_* is the labeled filling weight (0.31 g).

#### 3.5.3. Capsule Dissolution Evaluation

The dissolution determination method was adapted from a previously validated published study [51], in which the methodological verification, including specificity, linearity, precision, accuracy and solution stability, had been fully completed. The verified method meets the requirements of quantitative analysis and Pharmacopoeia specifications and was therefore applied to the in vitro dissolution evaluation of Guizhi Fuling capsules in this work.

Dissolution testing was performed using an RC806D dissolution tester (Best Instrument Technology Co., Ltd., Beijing, China) following the paddle method (Method 2) described in General Chapter 0931 Dissolution and Release Tests of the ChP 2025 Edition, Volume IV [54]. A 1000 mL dissolution vessel was used, and the paddle was positioned centrally at a fixed height of 25 mm above the vessel bottom. The paddle rotating speed was set at 100 rpm, 900 mL of deionized water served as the dissolution medium, and the water bath temperature was controlled at 37 ± 0.5 °C. Prior to testing, 0.45 μm microporous membranes were pre-wetted with deionized water to prevent component adsorption.

Three batches of TSWG-made Guizhi Fuling capsules and commercial capsules were tested, with three parallel replicates for each group. Each capsule was placed in a sinker, immersed in pre-warmed dissolution medium, and the timing was started immediately. Nine sampling time points were set within 30 min: 1, 3, 4, 5, 7, 9, 12, 15 and 30 min. At each time interval, 10 mL of medium was withdrawn at the standard sampling height, filtered through a pre-wetted 0.45 μm membrane, and an equal volume of preheated deionized water was replenished immediately to maintain constant volume. The subsequent filtrate was collected as the test solution. With a large volume of dissolution medium and constant volume maintenance, sink conditions were maintained during the experiment.

A self-control mode was adopted in this assay. The filtrate sampled at 60 min was taken as the reference solution, which conformed to the rapid-release property of commercial gastric-soluble capsules. The absorbance was measured at 274 nm using a TU-1900 UV–Vis spectrophotometer (Beijing Puxi General Instrument Co., Ltd., Beijing, China), and cumulative dissolution curves were plotted accordingly. The cumulative dissolution percentage was calculated by Equation (12):

(12) $$Q_{j}(\%)=\frac{m_{i}\times A_{i}\times w_{i}}{m_{s}\times A_{s}\times w_{s}}\times 100$$

where *m_i_* is the dilution factor of the test solution, *m_s_* is the dilution factor of the reference solution, *A_i_* is the absorbance of the test solution, *A_s_* is the absorbance of the reference solution, *w_i_* is the content mass of the tested capsule, and *w_s_* is the content mass of the reference capsule.

### 3.6. Data Analysis

ANOVA, regression modeling and multi-response optimization for the DoE data were performed using Design-Expert 13.0 software, with a significance level set at *p* < 0.05. All particle property characterization tests were performed in triplicate (*n* = 3). 95% confidence intervals were calculated based on the replicated characterization data, and residual diagnostics were conducted to evaluate the fitness of regression models. Principal component analysis was adopted to explore the correlations between granule quality attributes. All data were preprocessed by mean centering and unit variance scaling before analysis, and model performance was evaluated via *R*^2^*X* and cumulative variance contribution rate. PCA was conducted using SIMCA 13.0 (Umetrics, Umeå, Sweden).

## 4. Conclusions

This study aimed to achieve efficient continuous granulation for hard capsules filled with cohesive NPPs. Notably, TSWG was introduced into this research field for the first time, addressing an application gap in the continuous manufacturing of NPP-based capsule preparations. By adopting the MPEID methodology, synergistic optimization of screw configuration and critical process parameters was achieved, resulting in improved granule size uniformity and operational stability. The reliability of the optimized process was confirmed through comprehensive particle-quality assessment and multivariate statistical analysis, and all core quality indicators met predefined pharmaceutical production specifications. Moreover, the established equivalent formulation strategy mitigated the limitation of scarce raw material availability during early development, thereby accelerating process iteration. In addition, 3D printing enabled low-cost fabrication of customized, non-standard screw elements, offering a preliminary technical reference for laboratory research and translational exploration of NPP continuous manufacturing.

Nevertheless, the MPEID methodology has limitations: it was developed and validated using a botanical drug formulation, and its applicability to synthetic API systems remains to be further validated. Additionally, the 3D-printed screw elements in this study were optimized for laboratory-scale development, and their adaptation to industrial equipment warrants further investigation. While the approach has limitations—particularly the formulation dependency of the RSM model, which may lead to deviation in torque prediction for the equivalent formulation—it nevertheless provides preliminary evidence of processability under TSWG operating conditions. The resin-based 3D-printed screw elements were designed solely for rapid prototyping and laboratory-scale process exploration. Due to insufficient hardness and wear resistance, conventional photopolymer resins cannot meet the material requirements for GMP-regulated pharmaceutical manufacturing. However, the digital screw-configuration workflow developed in this work may be transferable to industrial production, provided that further validation is conducted. The optimized screw configuration can be manufactured using GMP-compliant materials, such as medical-grade stainless steel, followed by scale-up validation and long-term process robustness studies.

Future work will integrate PAT tools (e.g., near-infrared spectroscopy and real-time particle-size monitoring) to enable improved process control. Advanced modeling approaches (e.g., machine learning) can be adopted to improve prediction accuracy, shifting process development from empirical optimization toward digital-twin-driven intelligent optimization. In addition, scale-up verification trials and content-uniformity tests will be conducted to evaluate the industrial applicability and practical feasibility of the optimized TSWG process.

## Figures and Tables

**Table 1:** Combinations of non-critical parameters based on particle size and flowability performance.

Runs	*A*: Pre-Kneading CE’s Lead (mm)	*B*: Post-Kneading CE’s Lead (mm)	*C*: KE’s Position (mm)	*F*: Screw Speed (rpm)
R2	45	36	22	200
R5	36	18	22	100
R9	36	36	22	100
R10	36	18	22	200

**Table 2:** The regression equation of the response surface quadratic model and its ANOVA result.

Response Variable	Regression Equation		*R* ^2^	*R_aj_*^2^ *	*R_pre_*^2^ *	*p* (Lack of Fit)	*PRESS* *
*Y_1_*: *Yield_med_*	=51.17 − 2.100*D* − 3.910*E* − 3.680*G* + 6.650*DE* − 7.050*DG* − 5.660*EG* − 7.870 *D^2^* + 8.830*G^2^*	(1)	0.9735	0.9381	0.7324	0.1026	36.21
*Y_2_*: ${\mathrm{cosine}}_{\mathrm{GSD}}$	=1.006 + 2.208 × 10^−4^*D* − 1.612 × 10^−3^*E* − 0.07035*G* + 5.450 × 10^−5^*DE* + 1.968 × 10^−3^*DG* + 0.01470*EG* − 1.095 × 10^−5^*D*^2^ − 8.889 × 10^−4^*E*^2^ − 0.1003*G*^2^	(2)	0.9767	0.9349	0.6575	0.1351	4.311 × 10^−6^
*Y_3_*: *IC*	=9798 × 10 − 433.0*D* − 5469*E* − 4.113 × 10^3^*G* + 97.36*DE* + 6.871*G^2^*	(3)	0.9463	0.9164	0.7848	0.4023	2.707 × 10^7^
*Y_4_*: *Torque*	=2.914 − 0.2999*D* + 1.037*E* + 14.26*G* − 4.569 × 10^−2^*DE* − 0.1466*DG* + 1.258 × 10^−2^*EG* + 6.066 × 10^−3^*D*^2^ + 0.2273*E*^2^ − 16.08*G*^2^	(4)	0.9824	0.9528	0.7940	0.4275	0.4319

* *R_aj_*^2^: adjusted *R*^2^; *R_pre_*^2^: predicted *R*^2^; *PRESS*: predicted residual sum of squares.

**Table 3:** Predicted optimal process parameters and measured values of TSWG validation experiments ($\overset{-}{x}$ ± *s*, *n* = 3).

Optimal Process	Materials	*D*_a_ (g/cm^3^)	*D*_c_ (g/cm^3^)	*D*_10_ (μm)	*D*_50_ (μm)	*D*_90_ (μm)	*Span*
*SA* = 45°, *thick* = 2 mm, *L/S* = 0.32	F1	0.47 ± 0.02	0.65 ± 0.03	3.66 ± 0.39	33.64 ± 4.30	137.55 ± 5.89	3.91 ± 0.29
	CEP	0.44 ± 0.00	0.55 ± 0.00	15.75 ± 0.35	66.51 ± 5.77	208.98 ± 11.11	2.61 ± 0.10

**Table 4:** Predicted and measured response values of granule properties and process responses in TSWG validation experiments.

Material Code	Predicted Value			Measured Value ($\overset{-}{x}$ ± *s*, *n* = 3)			*RE* (%)		
	${\mathrm{cosine}}_{\mathrm{GSD}}$	*IC*	*Torque* (Nm)	${\mathrm{cosine}}_{\mathrm{GSD}}$	*IC*	*Torque* (Nm)	${\mathrm{cosine}}_{\mathrm{GSD}}$	*IC*	*Torque*
F1	0.9970	24.67	1.38	0.9939 ± 0.0011	26.67 ± 1.05	1.94 ± 0.00	−0.31	+7.49	+28.76
CEP	0.9970	24.67	1.38	0.9994 ± 0.0001	20.39 ± 0.31	1.48 ± 0.06	+0.24	−20.97	+6.62

**Table 5:** The physical parameters for characterizing powders.

Incidence Factor	Parameters	Acronyms
Fundamental property	True density	*D*_t_
	Particle size	*D*_10_, *D*_50_, *D*_90_
Dimension	Bulk density	*D*_a_
	Tapped density	*D*_c_
	Porosity	$\varepsilon_{p}$
	Solid fraction	*SF_p_*
Compressibility	Inter-particle porosity	*Ie*
	Carr’s index	*IC*
	Cohesion index	*Icd*
Flowability	Hausner ratio	*IH*
	Angle of repose	*AOR*
	Flow time	*t″*
Stability	Moisture content	*%HR*
	Hygroscopicity	*%H*
Homogeneity	Particle size less than 50 μm	*%pf*
	Homogeneity index	*Iθ*
	Particle size distribution	*Span*

**Table 6:** Factors and levels for the Plackett–Burman design.

Factor Code	Factor Name	Level		Response Variable
		Low (−1)	High (+1)	
A	Lead of CEs in the first conveying zone (mm)	36	45	Medium particle fraction (*Yield_med_*, *Y_1_*, *%*), ${\mathrm{cosine}}_{\mathrm{GSD}}$ (*Y_2_*), *IC* (*Y_3_*), Torque (*Y_4_*, Nm)
B	Lead of CEs in the second conveying zone (mm)	18	36	
C	Position of the kneading element (mm)	22	175	
D	*SA* of the kneading discs (°)	30	60	
E	Thickness of the kneading discs (mm)	1.8	5	
F	Screw speed (rpm)	100	200	
G	*L/S* ratio	0.16	0.32	

**Table 7:** Factors and levels for the Box–Behnken design.

Factor Code	Factor Name	Value Type	Level		
			Low (−1)	Mid (0)	High (+1)
D	*SA* of the kneading discs (°)	Discrete	30	45	60
E	Thickness of the kneading discs (mm)	Continuous	1.8	3.4	5.0
G	*L/S* ratio	Continuous	0.16	0.24	0.32

## Data Availability

The data presented in this study are available in Supplementary Materials.

## Supplementary Materials

The following supporting information can be downloaded at: https://www.mdpi.com/article/10.3390/ph19060921/s1, Supplementary Materials S1: Table S1: PC1/PC2 scores of excipient powders (PCA Model 1) and Euclidean distances between excipients and CEP; Table S2: The Plackett–Burman experimental design for the screw elements and process parameters of twin-screw wet granulation; Table S3: The Box–Behnken experimental design for the screw elements and process parameters of twin-screw wet granulation; Table S4: The physical properties of granules and the response result. Supplementary Materials S2: Figure S1. The loading plot based on PC1 and PC2 of PCA Model 1; Figure S2. The loading plot based on PC3 and PC4 of PCA Model 1; Figure S3. Residual plots for four response models. (A–C) Normal probability plot, residuals versus predicted values, and residuals versus run order of Model *Y1* (*Yieldmed*); (D–F) Corresponding residual plots of Model *Y2* (*cosineGSD*); (G–I) Corresponding residual plots of Model *Y3* (*IC*); (J–L) Corresponding residual plots of Model *Y4* (*Torque*).

## Abbreviations

The following abbreviations are used in this manuscript:

ANOVA	Analysis of variance
BBD	Box–Behnken design
CE	Conveying element
CEP	Guizhi Fuling compound extract powder
CMAs	Critical material attributes
CPPs	Critical process parameters
CQAs	Critical quality attributes
DOE	Design of experiments
GSD	Granule size distribution
L/D	Length-to-diameter
KE	Kneading element
LLCE	Long lead conveying element
LSL	Lower specification limit
MCC	Microcrystalline cellulose
MLCE	Medium lead conveying element
MPEID	Material–process–equipment integrated design
PCA	Principal component analysis
PBD	Plackett–Burman design
PGS	Pregelatinized starch
QbD	Quality by design
SC	Screw configuration
SLCE	Small lead conveying element
TSWG	Twin-screw wet granulation
USL	Upper specification limit
