# Supplementary material for: A Material–Process–Equipment Integrated Design Method for Accelerating the Process Development of Twin-Screw Wet Granulation
Source: Pharmaceuticals (Basel). 2026 Jun 11;19(6):921. doi: 10.3390/ph19060921 (PMC13304837; doi:10.3390/ph19060921)
Supplement: Supplementary file 1 [file pharmaceuticals-19-00921-s001.zip › Supplementary Material S2.pdf]

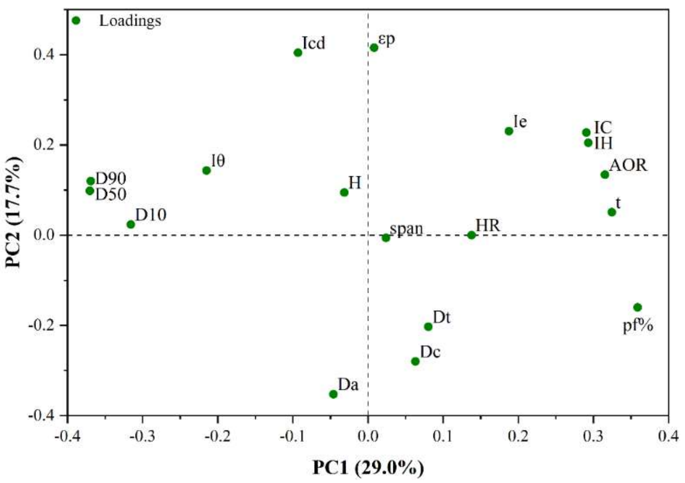

Figure S1. The loading plot based on PC1 and PC2 of PCA Model 1.

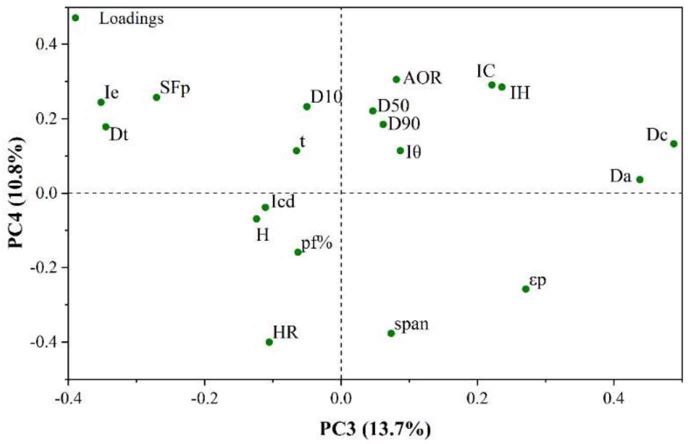

Figure S2. The loading plot based on PC3 and PC4 of PCA Model 1.

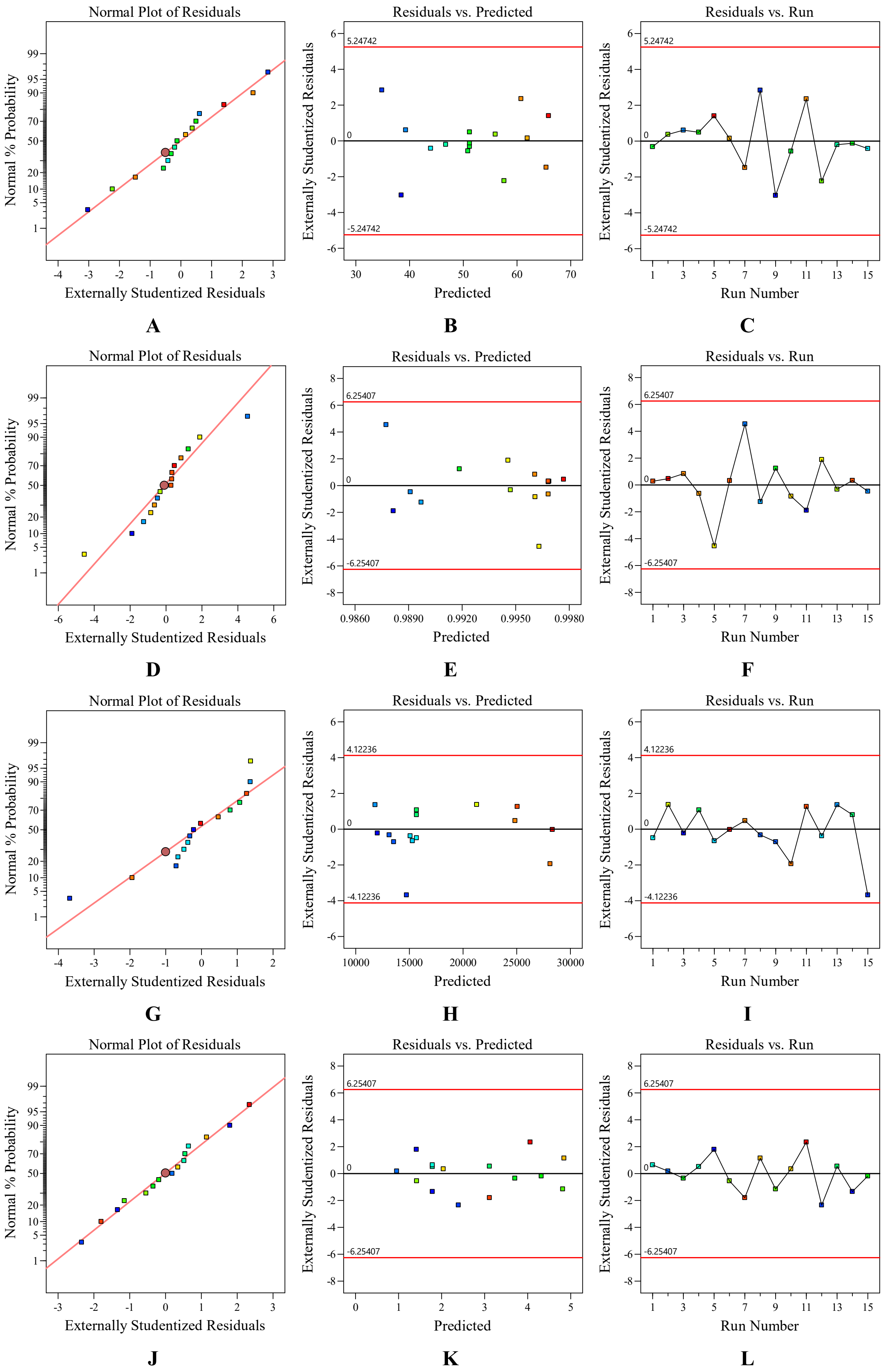

Figure S3. Residual plots for four response models. (A–C) Normal probability plot, residuals versus predicted values, and residuals versus run order of Model *Y1* ($Yield_{med}$); (D–F) Corresponding residual plots of Model *Y2* ($cosine_{GSD}$); (G–I) Corresponding residual plots of Model *Y3* (*IC*); (J–L) Corresponding residual plots of Model *Y4* (*Torque*).
